# Mitochondrial metagenomics: letting the genes out of the bottle

**DOI:** 10.1186/s13742-016-0120-y

**Published:** 2016-03-22

**Authors:** Alex Crampton-Platt, Douglas W. Yu, Xin Zhou, Alfried P. Vogler

**Affiliations:** Department of Life Sciences, Natural History Museum, London, SW7 5BD UK; Department of Genetics, Evolution and Environment, University College London, Gower Street, London, WC1E 6BT UK; State Key Laboratory of Genetic Resources and Evolution, Kunming Institute of Zoology, Kunming, Yunnan Province 650223 China; School of Biological Sciences, University of East Anglia, Norwich Research Park, Norwich, Norfolk NR4 7TJ UK; China National GeneBank, BGI-Shenzhen, Shenzhen, Guangdong Province 518083 China; Department of Life Sciences, Silwood Park Campus, Imperial College London, Ascot, SL5 7PY UK

**Keywords:** Shotgun sequencing, Illumina, Biodiversity, Phylogenetics, Community ecology, Genome assembly

## Abstract

‘Mitochondrial metagenomics’ (MMG) is a methodology for shotgun sequencing of total DNA from specimen mixtures and subsequent bioinformatic extraction of mitochondrial sequences. The approach can be applied to phylogenetic analysis of taxonomically selected taxa, as an economical alternative to mitogenome sequencing from individual species, or to environmental samples of mixed specimens, such as from mass trapping of invertebrates. The routine generation of mitochondrial genome sequences has great potential both for systematics and community phylogenetics. Mapping of reads from low-coverage shotgun sequencing of environmental samples also makes it possible to obtain data on spatial and temporal turnover in whole-community phylogenetic and species composition, even in complex ecosystems where species-level taxonomy and biodiversity patterns are poorly known. In addition, read mapping can produce information on species biomass, and potentially allows quantification of within-species genetic variation. The success of MMG relies on the formation of numerous mitochondrial genome contigs, achievable with standard genome assemblers, but various challenges for the efficiency of assembly remain, particularly in the face of variable relative species abundance and intra-specific genetic variation. Nevertheless, several studies have demonstrated the power of mitogenomes from MMG for accurate phylogenetic placement, evolutionary analysis of species traits, biodiversity discovery and the establishment of species distribution patterns; it offers a promising avenue for unifying the ecological and evolutionary understanding of species diversity.

## Background

DNA sequencing has been used widely for the study of biodiversity since the beginning of the PCR revolution in the late 1980s that permitted the analysis of targeted gene regions across taxa and populations [1, 2]. These studies produced a huge resource that includes sequence data for several hundred thousand species, in particular for rRNA and mitochondrial genes, including the *cox1* (or COI) ‘barcode’ marker [3, 4]. At the same time, our knowledge of Earth’s species diversity is far from complete [5], and although DNA methods can speed up the taxonomic process [3, 6], the gain has only been moderate for many species-rich groups and complex ecosystems because of the need for labour-intensive individual DNA extraction, PCR, and Sanger sequencing. This has limited the scope of individual DNA-based studies and thus the large-scale study of ecological and evolutionary processes.

These processes act at various spatial and temporal scales, and diversity is studied at multiple levels of organization, from genes to populations, species, communities and regional species pools. However, the various subdisciplines of ecology and evolution do not generally span these different levels, particularly in insects, because of constraints imposed by high species diversity and abundance. A more integrative approach to understanding the pattern of biological diversity, and the driving processes thereof, will require the use of universal character systems. Such a system should be informative at multiple hierarchical levels, from within-population variation to species boundaries and deep phylogenetic relationships. The approach that we describe here builds on the long-standing research that has generated mitochondrial sequence data to study virtually any question in ecology and evolutionary biology, and across organizational levels. For example, mitochondrial DNA (mtDNA) has been the backbone of phylogeography [7], and the *cox1* barcode is equally prominent in DNA-based species identification and species delimitation. In addition, mtDNA is widely used in phylogenetics, from the generation of very large trees at species level [8] to studies of relationships at deep hierarchical levels [9].

The short mitochondrial sequences generated by PCR have frequently been found to hold insufficient information for studies of population biology, biodiversity and, in particular, phylogenetics. Meanwhile, full mitochondrial genomes have been difficult to obtain until recently, requiring a tedious process of long-range PCR amplification followed by primer walking (e.g. [10]). Such processes are poorly suited to high-throughput biodiversity applications, and they also limit the viability of mito-phylogenomics. Several of the early failures of mitogenomics may, in part, be a byproduct of this production bottleneck, as denser taxon sampling [11, 12] and the use of more complex likelihood models [13] is increasingly demonstrating the utility of mitochondrial genomes at various hierarchical levels. The advent of high-throughput sequencing (HTS) is now removing some of the practical constraints, allowing both cheaper sequencing of mitogenome fragments obtained by PCR and the *de novo* assembly of mitogenome sequences from short reads produced by increasingly economical shotgun sequencing of genomic DNA [14].

These developments also relate to the study of biodiversity, as genomic DNA extracted in bulk from specimen mixtures - such as those obtained by mass trapping of invertebrates [15, 16] - or environmental DNA (eDNA) [17] can now be subjected to shotgun sequencing, genome assembly, and bioinformatic selection of the marker of interest - either the barcode region specifically or the whole mitogenome of numerous species simultaneously. Low-coverage shotgun sequencing of total DNA generates reads from all parts of the genome, but only the high-copy-number elements and repeat regions are present in sufficient quantities to permit assembly into longer contigs, in a process referred to as ‘genome skimming’ [18]. Thus, rRNA, histone genes and mitochondrial (and other plastid) genomes are assembled preferentially because of their high copy number per nuclear genome, providing a natural enrichment. For example, mitochondrial DNA is estimated to be present in 200 copies per nuclear genome in *Drosophila melanogaster* [19].

‘Mitochondrial metagenomics’ (MMG) [20] (also called ‘mito-metagenomics’ [21]) is a specific form of metagenome skimming [22], targeting the mitochondrial fraction of bulk specimen sequencing. MMG represents a simple and economical method for the high-throughput generation of mitogenome sequences for systematics, and it is particularly relevant to the study of natural arthropod communities, exploiting the proven utility of whole mitochondrial genomes in studies of population genetics, species delimitation, and phylogenetics. In the following sections, we describe procedures for extracting mitogenomes at a large scale and the methodological challenges of working with specimen mixtures of various kinds. We also present some early results in the study of insect communities and highlight the immediate targets for further development.

## Review

### A framework for applying mitochondrial metagenomics

Mitochondrial metagenomics is conducted on pooled DNA from numerous species, i.e. specimens are not individually indexed, and relies on the correct reconstruction of orthologous sequences *in silico*. Following the introduction of HTS, it was established that multiple mitogenomes can be assembled correctly in a single sequencing run for dozens of species combined, initially using mixtures of long-range PCR amplicons and reads of up to 450 bp from the 454 sequencing platform [23], and later backed up by simulation studies [24]. Subsequently, the larger volume of reads produced by Illumina sequencers made sequencing total genomic DNA of specimen mixtures feasible without the use of PCR [25], as first suggested by Taberlet et al. [26].

**Fig. 1:**
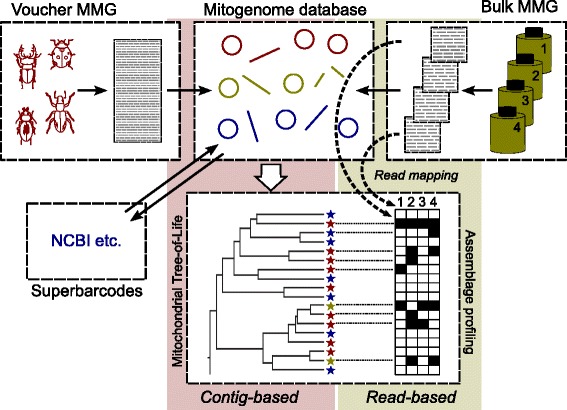
A schematic diagram of mitochondrial metagenomics. The central panel (*red*) represents the ‘contig-based’ analyses, using a database of complete (*circles*) or partial (*lines*) mitochondrial contigs. These are derived from one or more sources: sequencing of taxonomically chosen specimens and/or representative specimens from an ecological study (Voucher MMG; *left panel*); direct assembly of ecological bulk samples (Bulk MMG; *right panel*); external databases containing identified mitogenome sequences (*superbarcodes*), such as NCBI. Specimens for voucher and bulk MMG are shotgun- sequenced in mixtures, assembled with standard assembly pipelines, annotated for each gene, and assigned to known species through matches with *cox1* barcodes or other mitochondrial sequences from well-identified specimens where applicable. The ‘contig-based’ analysis concludes with a phylogenetic analysis, with the tree updated as new data become available in an iterative process. This set of mitogenomes can then be used as a reference for a ‘read-based’ analysis (*green panel*). Here the presence and possible abundance of a given species in the local assemblage is determined by mapping reads from ecological bulk samples against the mitogenome database (*dashed arrows*). The knowledge of the phylogenetic tree provides an evolutionary perspective to all species in the study

The MMG workflow (Fig. 1) starts with a pool of genomic DNA from multiple specimens that is shotgun sequenced, currently using Illumina technology. Specimens can either be a taxonomically chosen set that is mixed together deliberately (hereafter called ‘voucher MMG’) - for example, because of their interest to a particular phylogenetic or ecological study - or they may come from mass-trapped specimen ‘soups’ [16] that are sequenced directly (hereafter called ‘bulk MMG’). For voucher MMG (Fig. 1, top left), DNA from each specimen is separately extracted and aliquots are pooled in roughly equal concentrations before shotgun sequencing. The resulting short reads are assembled into full-length contigs using standard genome assembly software. Mitogenome contigs are associated with their source specimens by matching against an *in silico* ‘bait’ sequence [23] from PCR-amplified individual DNA samples. Often this will be the *cox1* barcode region (*cox1*-5′), although *cox1*-3′, *cob*, *nad5*, and *rrnL* have also been used. Mitogenomes from voucher MMG thus become a ‘superbarcode’ reference dataset tied to physical specimens with taxonomic information. Bait sequences may be available already for some or all of the pooled species, obviating the need for additional Sanger sequencing, and voucher DNAs may be obtained for MMG from existing barcoding studies, simplifying the process of building a superbarcode library.

In the alternative approach of bulk MMG (Fig. 1, top right), DNA is mass-extracted from a specimen ‘soup’ prior to shotgun sequencing and contig assembly, producing multiple mitogenomes or portions thereof. This avoids the effort of making a reference dataset of taxonomically curated voucher specimens that can be tied to the mitogenomes. However, avoiding this step poses new challenges in how to use the resulting information without proper taxonomic or phylogenetic context, and how to deal with the increased analytical complexity resulting from uneven species biomass and genetic variation. Sequences generated by bulk MMG are usually not identifiable to a species because of the incompleteness of existing barcode databases. They can, however, at least be assigned to some taxonomic rank by comparison against the rapidly growing database of short mitochondrial sequences from fully identified specimens [27] and/or by incorporating the complete or partial mitogenomes into a larger phylogeny with existing superbarcodes (Fig. 1, bottom) [20]. Importantly, this phylogenetic placement provides a robust superfamily- or family-level identification even with low levels of superbarcode sampling, and the resolution of such identifications improves with increasing sampling density [20]. At the same time, contigs assembled from bulk MMG samples will be biased towards the recovery of the most abundant species (in the sense of high biomass) unless sequenced to great depth. However, locally or temporally rare species may be abundant in at least some samples or sufficiently abundant overall such that combining bulk MMG samples from multiple sites will generate a largely complete database of encountered species. This contrasts with voucher MMG where database completion is limited primarily by sampling effort.

Both voucher and bulk MMG focus on the assembly of mitogenome contigs to populate a reference database relevant to a particular study, and so we refer to these analyses collectively as the ‘contig-based’ approach to MMG. For ecological studies, once we have a reference database (even one constructed only from public databases) we can then apply what we call the ‘read-based’ approach to MMG. This is the extraction of biodiversity information from large numbers of bulk samples by shotgun sequencing and the mapping of the resulting reads to the mitogenome reference database (Fig. 1, middle) [21, 28]. No assembly is carried out, although in the initial phase these reads might have been independently used to assemble contigs for the reference database (via bulk MMG). Given that these reads are a largely unprocessed sample of the genomes in a mixture, they can be used to establish species occurrence in a sample with high sensitivity for species presence and even relative abundance (biomass) [28, 29].

The mitogenome coverage required for secure detection of species presence from read mapping is much lower (at least by a factor of ten) than that required for *de novo* assembly. Thus, read mapping of low-coverage sequencing data detects low-biomass/abundance species more reliably than does contig assembly [28]. In addition, there is a strong correlation between input species biomass and mapped read numbers [28]; species occurrence, biomass, extrapolated species richness, and community structure were all recovered with less error than in a metabarcoding pipeline when applied to bee communities [29]. MMG is therefore a strong candidate for processing the large numbers of specimens that are expected to be collected by long-term monitoring programmes (e.g. for pollinators [29]).

It may even be possible to skip the generation of reference mitogenomes altogether and instead map reads against a database of only DNA barcodes. Although barcode sequences represent a much smaller target for mapping, in cases where there has been sufficient investment in barcoding the fauna under study, or only a limited subset of encountered species are of interest, this may be an economical solution for ongoing monitoring. Gómez-Rodríguez et al. [28] found that 658-bp *cox1* barcodes can have almost as much species-detection power as full mitogenomes when used as a mapping target but, because of their ~20x shorter sequence length, greater sequencing depth is required for the same detection limit. However, the longer mitogenome sequences produced in the initial contig-based phase of MMG present several important advantages that make the additional effort worthwhile. The first is greater species-detection confidence: species that are truly present in a sample will produce reads that map across the whole mitogenome, whereas laboratory contaminants (stray PCR amplicons from unrelated experiments and tiny amounts of tissue) will map to only one or a few loci. This includes nuclear mitochondrial pseudogenes (numts), which are frequently co-amplified with the true mitogenome but rarely extend beyond a single gene and whose stoichiometry is linked to the nuclear copy number. Second, mitogenomes, unlike barcodes, contain considerable phylogenetic information that can be used to characterize phylogenetic community diversity and turnover (see below). Third, mapping to the whole mitogenome increases the likelihood of detection for low-biomass species [28] and the accuracy of relative biomass quantification with appropriate species-specific benchmarking [29].

### Methodological issues

To date, MMG has used the Illumina HiSeq and lower-volume MiSeq sequencers with similar success. Direct comparisons of studies performed on either platform are complicated by differences in sequencing strategy. However, both have produced mitochondrial reads in the range of 0.5 % [21] to 1.4 % [20] of the total sequence data. The sequenced libraries had an insert size of 250 bp in the former and 850 bp in the latter, and a second library with an insert size of 480 bp had a lower proportion of mitochondrial reads (1.1 %; [20]) and resulted in shorter mitochondrial and non-mitochondrial contigs than the longer insert size library [22]. Thus, there is some indication that insert size affects mitochondrial proportion. However, the sample in [21] covered a range of insect and non-insect groups, whereas that in [20] contained only beetles; thus some of the observed differences in mitochondrial proportion may be accounted for by taxon-specific differences in the proportion of mitochondrial DNA relative to the nuclear (including symbiont) fraction. Regardless, the low overall proportion of mitogenome reads raises a question about the total amount of sequencing needed for successful assembly and how this is affected by the pooling strategy. From the voucher MMG studies to date, long mitogenome sequences were assembled with variable efficiency, ranging from approximately 1–2 mitogenomes per Gb with the shorter reads of the HiSeq [21] to 10 mitogenomes per Gb of sequence data with the MiSeq [12, 28]. Success was substantially lower when no equalization of DNA concentration was made (i.e. bulk MMG) [28]. Nevertheless, even assembly of mixed bulk samples can be improved by reassembling contigs from multiple assemblers, producing, for example, 124 long mitogenome sequences from 17 Gb of MiSeq data (250 bp, paired-end reads), equivalent to approximately seven mitogenomes per Gb [20].

Assessment of assembly success is also complicated by the fact that the criteria for reporting a mitogenome sequence as being ‘nearly complete’ differs between studies, e.g. the requirement for a contig to cover a minimum of eight protein-coding genes in one study [12] versus ten in others [20, 28]. Crucially, the number of truly complete, i.e. circular, mitogenome sequences is rarely stated. However, it seems clear that the equalization of DNA concentrations (including simply adjusting for body size) and the removal of intraspecific diversity (by including only one individual per species) undertaken in voucher MMG greatly increases the success of assembly, compared with a pool of specimens with no such adjustments [28]. In addition, more data per mitogenome can be gathered if two or more partial but non-overlapping contigs can be shown to represent the same mitogenome. Short contigs derived from the same mitogenome can be identified by similarity to other available full-length mitogenomes [30], by using multiple baits obtained from a single voucher [12], or by phylogenetic placement in a tree obtained from more complete mitogenomes. In such trees these partial contigs usually appear as sister taxa or close relatives that are separated by zero internode distances (as they constitute non-overlapping sequences without characters differentiating them from each other), while also having roughly equal read coverage [31].

**Fig. 2:**
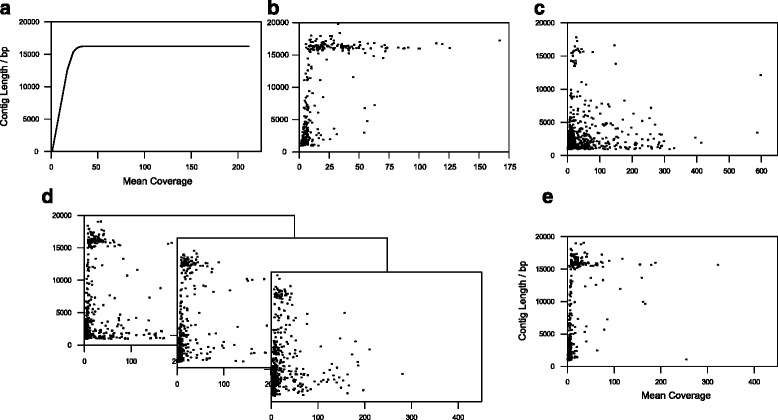
Coverage and mitochondrial contig length. **a** Coverage is approximately proportional to input species biomass; therefore, sequence contiguity (contig length) should increase with coverage, up to the minimum level of coverage required to obtain a full-length (~15–20 kb) mitogenome assembly. Increasing sequencing depth beyond this point is not cost-effective. **b** An example from [28], showing the mitochondrial contigs obtained in a reference set (one specimen per morphologically identified species, normalized for roughly equal DNA concentration based on body size), with read coverage calculated for each contig based on number of reads mapped. **c** Assembly from the same study [28] but made from mixed bulk specimens. **d** The use of different assemblers (left: IDBA-UD; middle: Newbler; right: Celera) on a mixed sample of rainforest beetles [20], showing fairly incomplete assembly even for mitochondrial contigs with high coverage. **e** Combining these three assemblies in Geneious to increase sequence contiguity resolves a large number of these cases but is not completely effective

That said, even where protocols have attempted to include the same amount of DNA per species, coverage of the resulting contigs has been uneven [12, 21]. These differences result from species-specific relative proportions of mitochondrial to nuclear DNA that are unknown *a priori* and therefore cannot be taken into account when generating pools for voucher MMG. For bulk MMG of biodiversity ‘soups’, estimating the amount of data required is even more challenging, because of both the highly uneven DNA contribution per species and the presence of intraspecific diversity. No study to date has been able to assemble a complete mitogenome sequence for all pooled species. Instead, plots of assembled mitogenome length as a function of coverage (estimated by read mapping) offer insight into the assembly behaviour of various MMG samples (Fig. 2). In any given dataset, contig length for each species is expected to increase with sequencing depth (up to the maximum sequence length of the full mitogenome, ~15–20 kb in insects), with the asymptote indicating the optimal sequencing depth for MMG (Fig. 2a; ~10x). Such a correlation was observed for the voucher MMG dataset of [28] that included only a single specimen per species (Fig. 2b), but not for bulk MMG samples comprising the same species, where higher coverage did not correlate with greater contig length, as evident from the presence of short contigs even where coverage was several 100x (Fig. 2c). Equally, three different assemblers showed a similar pattern of short, high-coverage contigs in the bulk MMG dataset of [20] (Fig. 2d), although contiguity was greatly improved by merging the three assemblies (Fig. 2e). This indicates that the assembly efficacy of voucher MMG can, to a large extent, be replicated for bulk MMG samples but requires extra steps for reassembly and adds complexity to the analysis.

Another consideration for the assembly procedure is the total volume of reads from which to conduct the assembly, which is a computationally costly step, particularly for complex samples. Assembly can be conducted on all reads or be limited to a subset filtered for similarity to existing mitogenome sequences, e.g. those available at the National Center for Biotechnology Information (NCBI). Filtering can be run via low-stringency (e.g. 1e-5) BLAST searches against a growing database of mitogenomes and can be expected to retain approximately 10 % of the reads for assembly [20, 21]. These searches are time-consuming but compensated for by greatly reduced data complexity, speeding up downstream assembly and mapping steps.

In the studies to date, a number of different assemblers have been used, but a rigorous assessment of the performance of a range of commonly used programs on a variety of voucher and bulk MMG datasets is still lacking. IDBA-UD [32], Celera Assembler [33], SOAPdenovo [34], SOAPdenovo-Trans [35], and Newbler [36] have been used most frequently, and all of these have successfully assembled long mitogenome sequences from MMG data. Generally, the assemblers produce closely similar contigs, although none of the existing assemblers has been found sufficient to extract the full information alone, and two or more assemblies have often been merged to increase the level of completion [12, 20, 21]. Automatic combining of contigs, e.g. using Minimus [37], tends to introduce errors, the source of which is difficult to trace. An alternative approach combining TGICL [38] reassembly with manual checks may be more successful [21] but has not yet been tested on complex samples. Iterative reassembly and manual curation in Geneious [39] have been used successfully for a complex sample, although the persistence of short, high-coverage contigs indicates that this process is not completely effective (Fig. 2e; [20]).

The development of an assembler specific to the problem of assembling multiple mitochondrial genomes from metagenome data is desirable. An existing mitogenome assembler, MITObim [40], has been used successfully for a range of taxa individually (e.g. [41–43]) and can assemble sequences for targeted species from metagenomic data (e.g. [44]). However, the utility of this program for bulk MMG, where the sample composition is not known *a priori*, has not yet been fully tested owing to the need for appropriate user-provided reference sequences, e.g. *cox1* barcodes or mitogenome sequences from close relatives of the target species. The procedure uses mapping of reads to the reference(s) to generate a new reference around the region of interest. These reads are matched to the new reference and assembled again using MIRA [45], which is repeated until the process reaches a stationary phase. Although this works effectively for single-species shotgun data, it is not designed to simultaneously assemble sequences from close relatives, and complex datasets are likely to require a large number of iterations and involve a more time-consuming mapping step. An alternative would be to seed the de Bruijn graph itself, possibly with short *cox1* sequences, but this has not been implemented and may be counterproductive where no prior sequence information for the taxa in the mixture are available. The key question here, and for MITObim, is the extent to which divergent references can be used as ‘generic’ mitochondrial seeds. In addition to an MMG-specific assembler and/or improvements in metagenome assembly algorithms in general, automatic identification of overlapping ends and the production of already-circularized contigs would be hugely beneficial and improve efficiency over current procedures which require manual checks for circularity [20, 21]. Geneious already supports circular assembly and works well with small, high-coverage datasets [46], but it is probably not practical for the more complex samples of typical MMG pools.

The final step of the process is the identification of homologous gene regions in the completed mitogenome sequence. This can be achieved using existing annotation software such as MITOS [47] or a reference sequence-based annotation pipeline [30]. For large datasets where annotation procedures are time-consuming, homologous regions can be extracted rapidly for phylogenetic analysis via BLAST, or by automated annotation of tRNA genes with COVE [48] and the extraction of intervening regions, which are then sorted into genes by mapping against a known reference [20]. The final contigs can be assessed for quality and corrected by comparing them with the original assemblies and by mapping back the reads [29]. However, for complex biodiversity samples the mapping step remains challenging with the software currently available, and the unevenness in the observed coverage within contigs is not necessarily indicative of incorrect assembly [20].

Assembling contigs from a mixture of species also carries the risk of chimeric sequences. These chimeras can be detected against known full or partial mitogenomes, where these are available, and by confirming that taxonomic assignments are consistent across the different genes in the assembly [21]. The latter method is, however, still limited by highly uneven taxonomic coverage in public databases across different mitochondrial genes [49]. Tests have also been done using multiple ‘bait’ sequences per source individual, which should each show the highest similarity to various parts of the same contig in the mixture. No exception to this expectation was found in nearly 100 mitochondrial assemblies of weevils [12]. In a bulk MMG experiment on whole communities with highly uneven DNA concentration and intraspecific variation, chimeras were detected against complete mitogenomes from the same species obtained via voucher MMG, but the proportion was very small (0.3 %) [28]. We conclude that under appropriate parameter settings, chimera formation is not a major concern in MMG. In addition, comparisons with conventional barcode sequences have revealed complete identity of the primary sequence in the assembled Illumina data, showing very good reliability of this next-generation sequencing technology [21, 31].

### The use of mitochondrial metagenomics in biodiversity studies

Metagenomic study of eukaryotic biodiversity based on the mitochondrial fraction is a new and rapidly expanding field. Most studies to date have not gone beyond the proof-of-concept stage and have been limited to insects, and a rigorous evaluation and optimization of key parameters is still lacking. However, the potential of MMG is already evident from these few studies, covering a range of questions from phylogenetics to community ecology. The number of specimens and samples that can be studied may be very large, becoming limited by the capacity of HTS and the availability of appropriate computing resources rather than by the cost of individual DNA extractions and Sanger sequencing. In addition, the growing availability of barcodes and mitogenomes from well-identified vouchers allows robust phylogenetic placement of newly assembled contigs and the study of taxa without expert taxonomic identifications at the outset. Linking species occurrences based on recovery of their mitogenome sequences between samples and studies, along with associated collection metadata, will rapidly build an image of their distribution and ecological associations as well. This process is unaffected by variation in taxonomic effort or knowledge, the taxonomic status of a particular species, or subsequent taxonomic revisions. However, the rapid growth of baseline distributional data built from MMG will require increased efforts to study the biology and ecology of poorly understood groups to ensure correct interpretation of the underlying biology.

Meanwhile, the quality of sequence identifications should be examined against validated public databases [50] and museum collections. Even though the DNA in museum specimens is degraded, the achievable read length is generally sufficient for assembling full or partial mitogenomes. Timmermans et al. [11] extracted DNA from pinned British butterflies collected mostly in the 1980s and 1990s, producing a mean mitochondrial read length of 167 bp and assembling contigs >10 kb for 10 of 35 specimens, and additional contigs of various sizes for most of the others. Even in the cases of assembly failure, most specimens still produced enough reads to cover the full length of the *cox1* barcode, which can be used to verify existing barcode records and match mitogenomes from future fresh collections back to the museum specimens for an authoritative identification. This will also allow existing biological knowledge and historical records based on morphology to be linked with sequenced mitogenomes and the growing database of species incidences derived from MMG. As a first step to maximizing the utility of MMG, all datasets and the associated metadata should therefore be published in a form that makes both the raw data and the assembled mitogenome contigs widely accessible and facilitates data mining.

MMG can provide the framework for unifying data from any kind of taxonomic or ecological study by grouping sequences at species or higher clade levels. For example, MMG on a sample of Coleoptera obtained by canopy fogging in the Bornean rainforest generated numerous mitogenomes [20]. By incorporating these sequences into an existing phylogenetic tree of major coleopteran lineages, a family-level placement could be established for most species in the sample without expert identification, which would have been extremely difficult, in any event, for a complex tropical assemblage. This approach can also place species known only from their barcodes into their phylogenetic context, which is not possible with barcode sequences alone. For example, among the Bornean mitogenomes, the *cox1* barcode extracted from one contig exhibited >98 % sequence similarity to an entry in the BOLD database for *Liroetiella antennata*, a species of Chrysomelidae (Galerucinae) that had been described from the Mount Kinabalu region of Sabah [51] and recently sequenced from Danum Valley [52], the same forest reserve from which the canopy sample had been obtained. The mitogenome study thus provided a solid phylogenetic placement for this newly described species relative to other lineages of Galerucinae, including several closely related species in the same sample. Over time, mitogenome data from multiple sources will inform each other and contribute to an ever more complete image of global biodiversity.

Exploiting taxon placement, the ‘predictive power’ of phylogenetics [53] also provides an evolutionary synthesis of species traits and reveals the factors driving the evolution and diversification of lineages. For example, Andújar et al. [31] used MMG to study communities of superficial- and deep-soil beetles. Six divergent lineages of Coleoptera were entirely confined to deep soil and, on inspection of the relevant specimens, these were found to be minute beetles exhibiting typical adaptations to a subterranean lifestyle, including the loss or reduction of eyes and a lack of body coloration. Thus, a major ecomorphological syndrome was detected from the phylogenetic placement of sequences and their circumstances of sampling alone. The MMG samples could be used to study phylobetadiversity (differences in phylogenetic composition of local assemblages) and thus provide a community-level perspective to evolutionary turnover that captures ecological processes in space and time [31]. The study found that species turnover among sampling sites was greatest in the deep soil layer, suggesting that dispersal is more restricted in deep soil than in the superficial (including leaf litter) layer, which has apparently resulted in greater species differentiation in deep soil. A key point is that this result was established for multiple independent lineages because MMG allowed whole assemblages to be studied, whereas a typical phylogenetic study would have focused on a single lineage, leaving open the question of whether the pattern was general.

With a growing database of mitogenomes (both well-identified superbarcodes and mitogenomes with an ecological context but only a higher-level identification) against which reads from local bulk samples can be mapped, distribution data will be rapidly accumulated without being biased by either the precision of identifications in any single study (as all studies will use common references), the focus on a subset of sampled species (as data for all sequenced species can be mined from the raw reads), or the life stage encountered (as life stages are linkable via their DNA). Access to reliable data on species richness and turnover for these groups may reveal biodiversity patterns that are currently unknown because of the focus on a limited set of easily observable taxa. This approach also supersedes tedious whole-community barcoding performed to establish the parameters determining community turnover. For example, the analysis by Gómez-Rodríguez et al. [28] of herbivore communities in Iberia used shotgun sequencing of 2600 specimens from ten communities and found evidence for increasing species turnover with geographic distance. This work had initially been done with Sanger-sequenced *cox1* barcodes [54], but the MMG data were much more quickly acquired and provided very similar conclusions about community composition.

Metagenomic sequencing could thus improve the study of biodiversity in two important dimensions: 1) by analyzing numerous species collectively and hence shifting the focus to the study of large species assemblages rather than individual species; 2) by characterizing all species in these assemblages simultaneously for their presence at particular sites, their phylogenetic position, their biomass (abundance), and possibly their within-species genetic variation. The approach can be conducted at any scale, from comparisons of local samples through to comparisons across biomes at a global level. In each case, the sequence data, via the phylogenetic tree obtained from mitogenomes, will readily place the encountered species in the context of other studies.

### Future prospects and next steps

One concern with the use of MMG may be the comparatively high cost of sequencing and bioinformatics required for data acquisition, including the ~99 % of reads corresponding to DNA that is not ultimately used. Unbiased enrichment of the mitochondrial fraction is therefore the most urgent target for future work if MMG is to be more widely used. It is straightforward to isolate intact mitochondria from live tissue by differential centrifugation, and very high concentrations of mitogenomes can be achieved in this way [55]. However, most samples in biodiversity surveys are obtained in preservation fluids, such as ethanol, in which mitochondria disintegrate. Separation at the DNA level, based on the lower specific weight of AT-rich mitogenomes in most arthropods, is possible using CsCl gradients [56], but conditions have to be optimized and the range of AT content of mtDNA of species in the mixture, and the great variation of nucleotide composition in the nuclear genomes, makes this an uncertain step. More promising are enrichment protocols using target enrichment with oligonucleotide probes designed based on known mitogenome sequences. This approach has already been successful in sequencing multiple mitogenomes from degraded DNA for a lineage of primates [57], although for the study of ‘unknown’ diversity, probes must capture a broader range of target molecules at greater genetic distance.

A recent study by Liu et al. [58] successfully enriched mitochondrial DNA for 49 taxa (mostly arthropods) from a previous study [29], using a probe array design based on mitochondrial protein-coding genes derived from more than 300 arthropod transcriptomes. The overall enrichment ratio was nearly 100x (from 0.47 to 42.5 % of total reads) and reads covering >80 % of the full mitogenome length were obtained for the majority of species tested, although the coverage rate was notably low in three of four Hymenoptera [58]. Tests indicated that regions of higher AT content and sequence dissimilarity to the probes were less likely to be captured effectively. Therefore, systematic tests of the efficiency of these enrichment procedures for varied taxonomic lineages and compositions, as well as optimization of probes, are needed for future studies. Crucially, for natural bulk samples this process should not skew the read proportions per species when compared with the unenriched sample. Additionally, although the degree of enrichment in the Liu et al. study was significant, coverage varied across the mitogenome and dropped to zero in places [58]. This is partly explained by the use of fragmented transcripts for probe design; however, variable sequence divergence between probes and targets along the length of the mitogenome will also contribute to gaps in coverage. Although this is not a major concern in the case of read-based MMG, such gaps may limit the assembly of long contigs from enriched samples and thus the success rate of contig-based MMG.

Several studies to date have shown a positive relationship between read numbers and proxy measures of biomass [12, 28–30]. Meanwhile, the microarray enrichment pipeline of Liu et al. [58] was found to maintain a strong correlation between input and output read numbers, suggesting that information on relative biomass could be retained in the enrichment step. In combination, these results indicate that MMG will provide useful biomass information (as a proxy for abundance) for ecological studies. However, such assessments may have to be carefully calibrated for each taxon, as the estimates are affected by the relative proportion of nuclear vs. mitochondrial DNA (because of variable nuclear genome size) and by the presence and abundance of gut microbes, which make a variable contribution to total read numbers [21, 29]. Thus, biomass estimates from MMG require prior tests of particular species of interest before it will be possible to monitor the relative biomass from read numbers. Current knowledge on the level of heterogeneity in mitochondrial sequence proportions within and between lineages is very limited as such tests are currently lacking in most organisms.

Another question relates to the use of MMG for assessing the intraspecific genetic variation represented by specimens in the mixtures. Assemblers are faced with the problem of building a single sequence from numerous short reads that contain slight variation due to sequencing errors, and this variation may be difficult to distinguish from true genetic variation. Thus far, assemblers have generally been observed to collapse the genetic variants present in a specimen mixture into a single sequence, effectively eliminating intraspecific variation. Genetic variation has been obtained by sequencing and assembly of separate samples, e.g. from multiple geographic sites or environments that may have different genotype compositions and therefore produce different consensus haplotypes [28, 31]. This property of the assembler limits an exact estimate from the contigs of the genetic variation in these mixed samples, and may in fact produce recombinant haplotypes, but this problem remains to be investigated. We already know that the problem is less severe for species-level divergences, as mitogenomes are usually assembled correctly for species within a genus [21, 28]. Hence a reliable estimate of intraspecific mitochondrial genetic diversity will probably be best obtained by mapping reads from natural samples to reference mitogenomes (either superbarcodes from voucher MMG or consensus contigs from bulk MMG) to call nucleotide variants, as the quality of the current Illumina technology appears to be adequate to generate secure single nucleotide polymorphism calls.

## Conclusions

In its short existence, MMG has been established as a powerful technique for biodiversity science and environmental management. The high sequencing volume per sample that can now be achieved economically is a perfect match for the needs of mixed-species analysis in complex biodiversity samples. Although mitochondrial genomes make up only a small proportion of the total sequence reads, they are the most useful marker to be extracted from these mixtures for this purpose. They are found in almost all eukaryotic species [9, 59], they have similar gene composition for easy establishment of orthology, and their genetic distances are fairly large in most metazoan animals and more uniform across genes than in the nuclear genome [60]. This distinguishes them from other high-copy markers, such as rRNA and histone genes, which contain highly conserved regions that hamper chimera-free assembly from species mixtures. MMG builds on and contributes to the large mtDNA databases that have been the mainstay of molecular phylogenetics [61, 62] and phylogeography [2], and more recently in DNA taxonomy with *cox1* barcodes [3]. With a growing, taxonomically curated reference set, it will be straightforward to identify many described, and previously encountered but unidentified, species in mass-trapped specimen samples by shotgun sequencing and simple similarity searches against this database. Full-length sequences, easily generated in huge numbers, can now exploit the power of mitogenomes to their full extent, for a synthesis of evolutionary and ecological research across various scales of biological organization. MMG can speed up the process of biodiversity discovery by integrating disparate biodiversity sequencing efforts for better assessment of the distribution and evolution of diversity in groups that are otherwise intractable to large-scale study. The current biodiversity crisis calls for strategies to streamline and unify efforts to catalogue the diversity and distribution of small-bodied eukaryotes. MMG is one such strategy, the longer-term utility of which will be determined by the success of efforts to tackle the remaining challenges highlighted in this review, and by the adaptation of existing MMG methods to ongoing developments in HTS technology.

## Abbreviation

MMG: mitochondrial metagenomics
